# The insulin resistance by triglyceride glucose index and risk for dementia: population-based study

**DOI:** 10.1186/s13195-020-00758-4

**Published:** 2021-01-05

**Authors:** Sangmo Hong, Kyungdo Han, Cheol-Young Park

**Affiliations:** 1 Department of Internal Medicine, Hanyang University, College of Medicine, 222, Wangsimni-ro, Seongdong-gu, Seoul, Republic of Korea; 2 Department of Statistics and Actuarial Science, Soongsil University, 369, Sangdo-Ro, Dongjak-Gu, Seoul, Republic of Korea; 3 Division of Endocrinology and Metabolism, Department of Internal Medicine, Kangbuk Samsung Hospital, Sungkyunkwan University School of Medicine, 29, Saemunan-ro, Jongno-gu, Seoul, 03181 Republic of Korea

**Keywords:** Alzheimer’s disease, Dementia, Dementia, vascular, Insulin resistance, TyG index

## Abstract

**Background:**

Insulin resistance is suggested to have negative effects on cognition; however, results from large population studies are lacking. In this study, the potential relationships between the triglyceride glucose (TyG) index, a simple surrogate marker of insulin resistance, and dementia were evaluated using a large-scale population dataset.

**Methods:**

This was a retrospective, observational, cohort study using data from the National Health Information Database from 2009 to 2015 and included 5,586,048 participants 40 years age or older. The TyG index was used as a measure of insulin resistance, and participants were divided into quartiles based on TyG index. The incidence of dementia was assessed using hazard ratios (HRs) estimated with Cox proportional hazard modeling.

**Results:**

During a median follow-up of 7.21 years, dementia was diagnosed in 142,714 (2.55%) participants. Alzheimer’s disease (AD) and vascular dementia (VD) were diagnosed in 74.3% and 12.5% of the participants. Multivariate-adjusted HRs for patients in the TyG index 4th quartile were higher for dementia (HRs = 1.14; 95% confidence interval [CI] 1.12–1.16), AD (HRs = 1.12; 95% CI 1.09–1.14), and VD (HRs = 1.18; 95% CI 1.12–1.23) compared with the 1st quartile of TyG index; however, this had a small effect size (Cohen’s *d* = 0.10, 0.08, and 0.13, respectively). These effects were independent of age, sex, smoking status, physical activity, body mass index, systolic blood pressure, and total cholesterol.

**Conclusion:**

In this large population study, TyG index was associated with an increased risk of dementia, including AD and VD, that was independent of traditional cardiovascular risk factors, although the effect size of the TyG index was small.

**Supplementary Information:**

The online version contains supplementary material available at 10.1186/s13195-020-00758-4.

## Introduction

Dementia, an aging-related disease, is a progressive neurodegenerative disorder clinically characterized by deterioration in memory, thinking, behavior, and the ability to perform everyday activities, and is one of the major causes of disability and dependency among older people worldwide. In addition, dementia is a social and economic burden, not only on patients with dementia, but also on their caregivers, families, and society at large. Dementia results from a variety of diseases and injuries such as Alzheimer’s disease (AD) or stroke that are primarily or secondarily associated with the brain. Among the types of dementia, AD is the most common, comprising up to 60–80% of dementia cases, and vascular dementia (VD) is the second most common, accounting for 10–20% of dementia cases [1].

Insulin resistance is a state of decreased responsiveness of target tissues to insulin, and a major feature of type 2 diabetes, glucose intolerance, hypertension, dyslipidemia, and cardiovascular disease. Some large population studies support an association between type 2 diabetes caused by insulin resistance and dementia [2–4]; although these studies were not able to establish the association between insulin resistance and dementia, because of the neurotoxic effects of hyperglycemia, a key feature of type 2 diabetes, a study with intranasal insulin administration reported improved memory and other cognitive functions in type 2 diabetes and suggested the association between insulin resistance and dementia [5, 6]. In several recent studies that involved both human and animal models, insulin resistance was suggested to have negative effects on cognition, particularly learning and memory [7, 8]; however, evidence for the association between insulin resistance and dementia from large population studies is lacking. Recently, the triglyceride glucose (TyG) index, a product derived from fasting levels of triglycerides and glucose as follows: ln[triglyceride (mg/dL) × fasting blood glucose (mg/dL)/2], has been suggested as a surrogate marker for the assessment of insulin resistance.

In the current study, the potential relationships between the TyG index and dementia were evaluated using a large-scale population dataset from the National Health Information Database (NHID).

## Methods

### Study design and participants

This was a national, observational, cohort study which followed 5,586,048 participants over an average of 7.21 years. We identified 7,183,262 participants 40 years of age or older who had participated in the National Health Screening program in 2009 from NHID. Among the participants, the following were excluded: 1,315,443 participants who took anti-diabetic medications or lipid-lowering medications, 267,277 participants who lacked complete data, and 14,494 patients with a history of dementia. Finally, the total number of eligible participants in the present study was 5,586,048. These were followed up until December 31, 2016.

### Study database

Data for the analysis were obtained from the NHID, a public database on health care utilization and health screening that contains sociodemographic and mortality information for the entire population of South Korea. The NHID contains data for the years between 2002 and 2016. The NHID, which is produced by the National Health Insurance Service, was launched by integrating 375 insurance associations in 2000, and provides longitudinal data for 97% of the Korean population, with linkage to the National Death Registry and the National Health Screening program [9, 10]. This latter program was initiated in 2009, and includes a medical interview and postural examination, chest X-ray examination, blood test (including fasting glucose and triglyceride levels), urine test, and dental screening, among others. Approval was obtained from the Institutional Review Board of Kangbuk Samsung Hospital for the study protocol (KBSMC 2018-01-036). Informed consent was waived by the board.

### Definitions of the TyG index and study outcomes (dementia)

The TyG index was calculated based on the equation derived in previous studies as follows: ln[triglyceride (mg/dL) × fasting blood glucose (mg/dL)/2] [11, 12]. Dementia was defined according to ICD-10 diagnosis codes for dementia (F00, G30, F01, F02, F03, G23.1, G31.0, G31.1, G31.82, G31.83, G31.88, and F10.7) concurrent with the prescription of anti-dementia medication. The anti-dementia medications included acetylcholinesterase inhibitors (rivastigmine, galantamine, or donepezil) or *N*-methyl-d-aspartate receptor antagonists (memantine), which are the most commonly used to treat dementia. AD was defined with ICD-10 codes (F00 and G30) concurrent with the prescription of anti-dementia medication. VD was defined with ICD-10 code (F01) concurrent with the prescription of anti-dementia medication. Participants without dementia during their follow-up were considered to have completed the study at the date of the first diagnosis of dementia or at the end of follow-up. The study population was followed up from baseline to the first diagnosis of dementia or until December 31, 2016.

### Clinical and laboratory measurements

All participants completed a questionnaire on their medical history, use of tobacco and alcohol, and exercise habits. Smoking status was categorized as non-smoker, ex-smoker, or current smoker; alcohol consumption was classified as non-drinker, moderate drinker (< 30 g per day), or heavy drinker (≥ 30 g per day); and regular exercise was defined as vigorous intensity exercise (three or more times per week) or moderate intensity exercise (five or more times per week). Low socioeconomic status was defined as income in the lowest 20%. Body mass index (BMI) was calculated as body weight (kg) divided by the square of the body height (m^2^). Blood pressure (BP) was measured using a standard procedure with a sphygmomanometer after rest for more than 5 min. Blood samples were collected after overnight fasting. Serum glucose, total cholesterol, triglyceride, high-density lipoprotein (HDL) cholesterol, and low-density lipoprotein (LDL) cholesterol were measured. Glomerular filtration rate was calculated using the four-variable Modification of Diet in Renal Disease Study equation [13]. Baseline comorbidities were identified as follows: hypertension (ICD-10 codes I10 to I13 or I15 and treatment with antihypertensive medications, systolic BP ≥ 140 mmHg, or diastolic BP ≥ 90 mmHg), type 2 diabetes (ICD-10 codes E11 to E14 and anti-diabetic drugs, or fasting glucose level ≥ 126 mg/dL), hyperlipidemia (ICD-10 code E78 with lipid-lowering agents, or serum total cholesterol ≥ 240 mg/dL), and chronic kidney disease (CKD; estimated glomerular filtration rate < 60 mL/min/1.73 m^2^).

### Statistical analyses

Baseline characteristics were analyzed using descriptive statistics. Categorical variables were described as frequencies and percentages. Continuous variables were described as means (± standard deviation, SD) for normally distributed data and geometric mean and 95% confidence interval (CI) for non-normally distributed data. Participant baseline characteristics for the TyG index quartiles were compared. Continuous variables were compared using one-way analysis of variance (ANOVA), and categorical variables were compared using the chi-square test. The follow-up duration based on TyG index quartiles was obtained. Based on TyG index quartiles, the incidence rate of dementia, AD, and VD was estimated using the total follow-up period for outcomes. Incidence curves were estimated using the Kaplan-Meier method, and the log-rank test was conducted. All outcomes were analyzed using Cox proportional hazards regression analysis while controlling for baseline covariates such as age, sex, smoking status, alcohol consumption, physical activity, low income, BMI, hypertension, and total cholesterol level which were significantly different between the quartiles in the baseline characteristics. To avoid co-linearity, hypertension but not systolic and diastolic BP, BMI but not waist circumference, and total cholesterol but not HDL cholesterol, and LDL cholesterol were used in the model fitting. We conducted sensitivity analyses that tested the interaction between subgroups by age, sex, current smoker, heavy drinker, regular exercise, diabetes, hypertension, dyslipidemia, body mass index, abdominal obesity, and quartiles of glucose and triglyceride to assess if the effect of TyG index for dementia differed between subgroups using Cox proportional hazards regression analysis adjusted for age, sex, smoking status, alcohol consumption, physical activity, income level, BMI, hypertension, and total cholesterol level. The validity of the proportional hazards assumption was assessed from the plots of LOG [−LOG (survival function)] versus LOG (follow-up time in years). The effect size was calculated using Cohen’s *d* with the following equation: *d* = ln (HR) × √ 6/π [14]. A two-tailed *p* value < 0.05 was considered statistically significant. Analyses were performed with SAS 9.4 (SAS Institute, Cary, NC, USA) and R programming, version 3.4.1 (The R Foundation for Statistical Computing, Vienna, Austria, http://www.R-project.org).

### Data availability

The NHID is a public open database. Access to the NHID can be obtained through the Health Insurance Data Service home page (https://nhiss.nhis.or.kr/bd/ab/bdaba000eng.do). Further inquiries on data use can be obtained by contacting the corresponding author.

## Results

### Baseline characteristics of study participants based on the TyG index

The baseline clinical and biochemical characteristics of the participants based on the TyG index quartiles are shown in Table 1. Among all participants, the TyG index across quartiles was positively associated with age, BMI, waist circumference, current smoking, alcohol consumption, systolic and diastolic BP, fasting glucose, total cholesterol, LDL cholesterol, triglyceride, high prevalence of type 2 diabetes, hypertension, dyslipidemia, and CKD (all *p* < 0.001). In addition, regular physical activity, low socioeconomic status, and HDL cholesterol were negatively associated with the TyG index across quartiles (all *p* < 0.001).

**Table 1:** Characteristics based on the TyG index quartiles of 5,586,048 participants obtained from the National Health Information Database between 2009 and 2015 in Korea

	TyG index quartiles				*p* value
	Q1	Q2	Q3	Q4	
TyG index					
Male	< 8.32	≥ 8.32, < 8.70	≥ 8.70, < 9.12	≥ 9.12	< 0.001
Female	< 8.05	≥ 8.05, < 8.41	≥ 8.41, < 8.79	≥ 8.79	< 0.001
n	2,109,016	2,109,379	2,105,801	2,108,850	
Age (years)	40.77 ± 13.3	44.08 ± 13.24	46.48 ± 13.12	48.49 ± 12.95	< 0.001
< 65	1,977,515 (93.76)	1,934,973 (91.73)	1,887,905 (89.65)	1,842,996 (87.39)	
≥ 65	131,501 (6.24)	174,406 (8.27)	217,896 (10.35)	265,854 (12.61)	
Sex					0.935
Male	1,174,379 (55.68)	1,173,923 (55.65)	1,172,194 (55.66)	1,173,998 (55.67)	
Female	934,637 (44.32)	935,456 (44.35)	933,607 (44.34)	934,852 (44.33)	
BMI (kg/m^2^)	22.13 ± 2.79	23.03 ± 2.98	23.9 ± 3.08	24.95 ± 3.13	< 0.001
≥ 25 kg/m^2^	310,449 (14.72)	500,595 (23.73)	705,893 (33.52)	987,536 (46.83)	< 0.001
Waist circumference (cm)	75.62 ± 8.29	78.24 ± 8.65	80.63 ± 8.67	83.49 ± 8.4	< 0.001
Male ≥ 90, female ≥ 85	139,015 (6.59)	257,122 (12.19)	400,791 (19.03)	623,333 (29.56)	< 0.001
Regular physical activity	1,142,254 (54.16)	1,111,265 (52.68)	1,086,109 (51.58)	1,050,834 (49.83)	< 0.001
Low socioeconomic status	582,805 (27.63)	559,430 (26.52)	546,658 (25.96)	542,423 (25.72)	< 0.001
Smoking status					< 0.001
Non-smoker	1,304,461 (61.85)	1,254,610 (59.48)	1,217,185 (57.8)	1,164,006 (55.2)	
Ex-smoker	284,472 (13.49)	296,684 (14.06)	299,196 (14.21)	286,796 (13.6)	
Current smoker	520,083 (24.66)	558,085 (26.46)	589,420 (27.99)	658,048 (31.2)	
Alcohol consumption					< 0.001
None/mild	1,012,048 (47.99)	1,037,179 (49.17)	1,051,377 (49.93)	1,037,392 (49.19)	
Moderate	986,225 (46.76)	943,026 (44.71)	903,865 (42.92)	868,558 (41.19)	
Heavy	110,743 (5.25)	129,174 (6.12)	150,559 (7.15)	202,900 (9.62)	
Systolic BP (mmHg)	117.24 ± 13.53	119.95 ± 14.08	122.49 ± 14.43	126.02 ± 14.93	< 0.001
Diastolic BP (mmHg)	73.22 ± 9.35	75 ± 9.63	76.6 ± 9.77	78.8 ± 10.03	< 0.001
Glucose (mg/dL)	87.16 ± 10.34	91.38 ± 11.41	94.66 ± 13.1	102.8 ± 24.02	< 0.001
Total cholesterol (mg/dL)	177.74 ± 29.97	189.18 ± 31.39	198.18 ± 32.91	209.72 ± 36.07	< 0.001
HDL cholesterol (mg/dL)	60.65 ± 15.48	57.44 ± 16.36	54.52 ± 17.67	50.44 ± 21.93	< 0.001
LDL cholesterol (mg/dL)	105.11 ± 28.07	113.45 ± 30.04	117.95 ± 31.77	113.9 ± 36.14	< 0.001
Triglyceride (mg/dL)	57.53 (57.51–57.55)	89.88 (89.85–89.9)	126.83 (126.78–126.87)	218.13 (218.02–218.25)	< 0.001
Type 2 diabetes	5628 (0.27)	18,042 (0.86)	43,375 (2.06)	180,205 (8.55)	< 0.001
Hypertension	222,942 (10.57)	334,170 (15.84)	450,707 (21.4)	630,060 (29.88)	< 0.001
Dyslipidemia	58,695 (2.78)	124,995 (5.93)	216,260 (10.27)	395,702 (18.76)	< 0.001
CKD	73,945 (3.51)	87,512 (4.15)	104,525 (4.96)	131,611 (6.24)	< 0.001

Data are mean (SD), *n* (%), or median (IQR). *TyG* triglyceride glucose, *BMI* body mass index, *BP* blood pressure, *HDL* high-density lipoprotein, *LDL* low-density lipoprotein, *CKD* chronic kidney disease

### Risk of incident dementia based on the TyG index quartiles

**Fig. 1:**
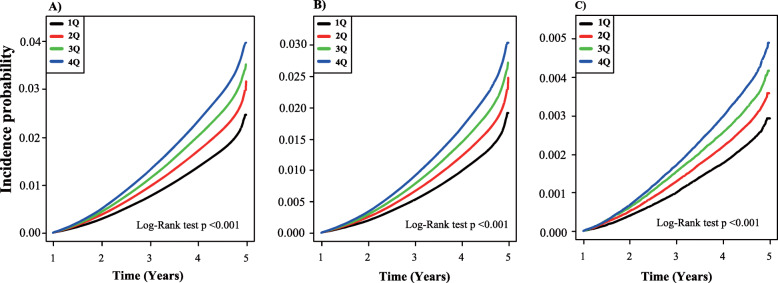
The cumulative incidence of dementia based on Kaplan-Meier. **a** Overall dementia. **b** AD. **c** VD during the average follow-up of 7.2 years based on the TyG index quartiles. Cumulative incidence probability is presented on the *y*-axis. Plots use different *y*-axis scales. The *p* value was calculated using the log-rank test. AD, Alzheimer’s disease; VD, vascular dementia; TyG, triglyceride glucose

Data from 5,586,048 participants (male 2,831,762 and female 2,754,286) were evaluated for an average of 7.21 years. During 40,271,758.61 person-years of follow-up, there were 142,714 incident cases of dementia (overall incidence of 2.55% or 3.54 cases/1000 person-years). Among 142,714 incident cases of dementia, 106,018 (74.3%) participants had AD and 17,800 (12.5%) had VD. Figure 1 shows the Kaplan-Meier curves for cumulative incidences of overall dementia for the TyG index quartiles. Participants in the 4th quartiles for the TyG index had the highest probabilities of developing incident dementia, and the probabilities decreased sequentially in the lower quartiles (log-rank *p* < 0.001).

The hazard ratio (HR) for dementia increased across TyG index quartiles (*p* for trend < 0.001): 1.23 (95% CI 1.22–1.26), 1.45 (95% CI 1.43–1.48), and 1.69 (95% CI 1.66–1.71) for the 2nd, 3rd, and 4th quartiles, respectively, compared with the 1st quartile (Table 2). In addition, age- and sex-adjusted HR for dementia increased across TyG index quartiles (*p* for trend < 0.001): 1.03 (95% CI 1.01–1.04), 1.05 (95% CI 1.04–1.07), and 1.11 (95% CI 1.09–1.13) for the 2nd, 3rd, and 4th quartiles, respectively, compared with the 1st quartile (Table 2). In a multivariate model adjusted for age, sex, smoking status, alcohol consumption, physical activity, income level, BMI, hypertension, and total cholesterol level, a progressive increase in the risk of dementia with TyG index levels in the 2nd, 3rd, and 4th quartiles was observed compared with the 1st quartile (*p* for trend < 0.001): HR 1.04 (95% CI 1.02–1.05), 1.07 (95% CI 1.05–1.09), and 1.14 (95% CI 1.12–1.16), respectively, with small effect sizes (Cohen’s *d* = 0.03, 0.05, and 0.10, respectively).

**Table 2:** Risk of dementia, AD, and VD based on the TyG index quartiles

TyG index	Events (*n*)	Duration (person-years)	Incidence rate (per 1000 person-years)	Unadjusted	Model 1	Model 2
All-cause dementia				HR (95% CI)	HR (95% CI)	HR (95% CI)
Q1	26,613	10,089,076.34	2.64	1 (ref.)	1 (ref.)	1 (ref.)
Q2	32,819	10,074,648.59	3.26	1.23 (1.22, 1.26)	1.03 (1.01, 1.04)	1.04 (1.02, 1.05)
Q3	38,606	10,066,646.27	3.84	1.45 (1.43, 1.48)	1.05 (1.04, 1.07)	1.07 (1.05, 1.09)
Q4	44,676	10,041,387.42	4.45	1.69 (1.66, 1.71)	1.11 (1.09, 1.13)	1.14 (1.12, 1.16)
*p* for trend				< 0.001	< 0.001	< 0.001
AD						
Q1	19,705	10,089,076.34	1.95	1 (ref.)	1 (ref.)	1 (ref.)
Q2	24,354	10,074,648.59	2.42	1.24 (1.21, 1.26)	1.02 (1.00, 1.03)	1.03 (1.01, 1.05)
Q3	28,661	10,066,646.27	2.85	1.46 (1.43, 1.48)	1.03 (1.01, 1.05)	1.05 (1.03, 1.07)
Q4	33,298	10,041,387.42	3.32	1.70 (1.67, 1.73)	1.07 (1.06, 1.09)	1.12 (1.09, 1.14)
*p* for trend				< 0.001	< 0.001	< 0.001
VD						
Q1	3341	10,089,076.34	0.33	1 (ref.)	1 (ref.)	1 (ref.)
Q2	4110	10,074,648.59	0.41	1.23 (1.18, 1.29)	1.08 (1.03, 1.13)	1.05 (1.01, 1.10)
Q3	4779	10,066,646.27	0.47	1.43 (1.37, 1.50)	1.14 (1.09, 1.19)	1.09 (1.05, 1.15)
Q4	5570	10,041,387.42	0.55	1.68 (1.61, 1.75)	1.25 (1.20, 1.31)	1.18 (1.12, 1.23)
*p* for trend				< 0.001	< 0.001	< 0.001

*AD* Alzheimer’s disease, *VD* vascular dementia, *TyG* triglyceride glucose index, *HR* hazard ratio, *CI* confidence interval

Model 1: age, sex

Model 2: age, sex, smoking status, alcohol consumption, physical activity, low income, body mass index (BMI), hypertension, total cholesterol level

### Risk of incident dementia in AD and VD subgroups based on the TyG index quartiles

The association between TyG index and the incidence of AD and VD was assessed. Among 142,714 incident cases of dementia, 106,018 participants had AD and 17,800 had VD. The incidence of AD and VD was 2.63 cases/1000 person-years and 0.44 cases/1000 person-years, respectively. Figure 1 shows the Kaplan-Meier curves for the cumulative incidences of AD and VD for the TyG index quartiles. The 4th quartile of the TyG index was associated with the highest probability of incident AD and VD, and the probability decreased sequentially in the lower quartiles (all log-rank *p* < 0.001). In the unadjusted model, the incidence of AD and VD also increased across TyG index quartiles. The HR for AD increased across TyG index quartiles: 1.24 (95% CI 1.21–1.26), 1.46 (95% CI 1.43–1.48), and 1.70 (95% CI 1.67–1.73) for the 2nd, 3rd, and 4th quartiles, respectively, compared with the 1st quartile (*p* for trend < 0.001, Table 2). HR for VD increased across TyG index quartiles: 1.23 (95% CI 1.18–1.29), 1.43 (95% CI 1.37–1.50), and 1.68 (95% CI 1.61–1.75) for the 2nd, 3rd, and 4th quartiles, respectively, compared with the 1st quartile in the unadjusted model (*p* for trend < 0.001, Table 2). In a multivariate model adjusted for age, sex, smoking status, alcohol consumption, physical activity, income level, BMI, hypertension, and total cholesterol level, the HR for AD increased across TyG index quartiles with small effect sizes (Cohen’s *d*): 1.03 (95% CI 1.01–1.05, *d* = 0.02), 1.05 (95% CI 1.03–1.07, *d* = 0.04), and 1.12 (95% CI 1.09–1.14, *d* = 0.08) for the 2nd, 3rd, and 4th quartiles, respectively, compared with the 1st quartile (*p* for trend < 0.001, Table 2). In the same model, the HR for VD increased across TyG index quartiles with small effect sizes (Cohen’s *d*): 1.05 (95% CI 1.01–1.10, *d* = 0.04), 1.09 (95% CI 1.05–1.15, *d* = 0.07), and 1.18 (95% CI 1.12–1.23, *d* = 0.13) for the 2nd, 3rd, and 4th quartiles, respectively, compared with the 1st quartile (*p* for trend < 0.001, Table 2).

### Sensitivity analysis: effects of clinical variables on the association between the TyG index and dementia

**Fig. 2:**
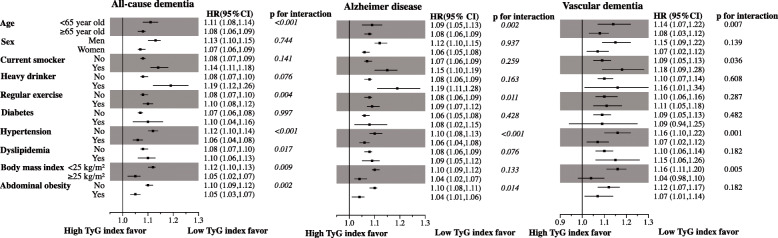
Risk of dementia in AD and VD subgroups based on the increasing TyG index quartiles with various clinical variables (stratified based on age, sex, smoking status, alcohol consumption, presence of diabetes, hypertension, and dyslipidemia, BMI, and presence of abdominal obesity). HR, hazard ratio; CI, confidence interval; AD, Alzheimer’s disease; VD, vascular dementia; TyG, triglyceride glucose; BMI, body mass index

To identify the potential effects of clinical variables on the relationship between increasing TyG index quartiles and incident dementia, subgroup analysis was performed for strata by age, sex, current smoker, heavy drinker, regular exercise, diabetes, hypertension dyslipidemia, BMI, and abdominal obesity with age, sex, smoking status, alcohol consumption, physical activity, income level, BMI, hypertension, and total cholesterol level in the adjusted model (Fig. 2). Regardless of the strata for dementia and cardiovascular risk factors (age, sex, current smoker, heavy drinker, regular exercise, diabetes, hypertension dyslipidemia, BMI, and abdominal obesity with age, sex, smoking status, alcohol consumption, physical activity, income level, BMI, hypertension, and total cholesterol level), increasing TyG index quartile was consistently associated with increased risk of dementia as well as AD and VD, except for developing VD in the population with diabetes or with BMI ≥ 25 kg/m^2^. The *p* value for interaction between subgroups was statistically significant for clinical variables which were associated with insulin resistance: age (*p for interaction* < 0.001), regular exercise (*p for interaction* = 0.004), hypertension (*p for interaction* < 0.001), dyslipidemia (*p for interaction* = 0.017), BMI (*p for interaction* = 0.009), and abdominal obesity (*p for interaction* = 0.002) in all-cause dementia; for age (*p for interaction* = 0.002), regular exercise (*p for interaction* = 0.011), hypertension (*p for interaction* < 0.001), and abdominal obesity (*p for interaction* = 0.014) in AD; and for age (*p for interaction* = 0.007), current smoker (*p for interaction* = 0.036), hypertension (*p for interaction* = 0.001), and BMI (*p for interaction* = 0.005) in VD. We also analyzed the risk of dementia by quartiles of triglycerides and glucose with a multivariate-adjusted model by age, sex, smoking status, alcohol consumption, physical activity, income level, BMI, hypertension, and total cholesterol level (Supplementary Table 1). The risk for all-cause dementia increased across triglyceride quartiles: 1.02 (95% CI 1.01–1.04), 1.06 (95% CI 1.04–1.08), and 1.10 (95% CI 1.09–1.12) for the 2nd, 3rd, and 4th quartiles, respectively, compared to the 1st quartile. The risk for AD increased in participants in the 3rd triglyceride quartile (HR = 1.05, 95% CI 1.03–1.07) and 4th triglyceride quartile (HR = 1.09, 95% CI 1.07–1.11) compared with the 1st quartile. The risk for VD increased in participants in the 3rd triglyceride quartile (HR = 1.08, 95% CI 1.03–1.13) and the 4th triglyceride quartile (HR = 1.13, 95% CI 1.08–1.19) compared with the 1st quartile. The risk for all-cause dementia and AD increased only in participants in the 4th glucose quartile (HR = 1.07, 95% CI 1.06–1.09, and HR = 1.07, 95% CI 1.05–1.09, respectively). However, the risk for VD was not statically different between glucose quartiles (Supplementary Table 1). We also conducted subgroup analysis by glucose and triglyceride quartile to assess the potential effects of triglyceride and glucose on the relationship between increasing TyG index quartiles and incident dementia in a multivariate-adjusted model (Supplementary Fig. 1). The increasing TyG index quartiles were significantly associated with an increasing risk for all-cause dementia, AD, and VD regardless of the triglyceride and glucose quartiles except for the 1st triglyceride quartile (which may be due to an exceedingly low incidence of dementia). There was no interaction between glucose quartiles [*p* for interaction = 0.392 (all-cause dementia), 0.757 (Alzheimer’s disease), and 0.294 (vascular dementia)]. However, there was an interaction between triglyceride quartiles in all-cause dementia (*p* for interaction = 0.001) and AD (*p* for interaction = 0.006), but not VD (*p* for interaction = 0.832).

## Discussion

In this longitudinal national representative cohort study, the risk of dementia based on the TyG index, a novel surrogate marker of insulin resistance, was assessed. Although the effect size was small, the findings from the present study showed that participants in the 4th quartile of the TyG index had a 14% increased risk for dementia compared with the 1st quartile and the risk of dementia increased across increasing TyG index quartiles (*p* for trend < 0.001), independent of age, sex, smoking status, alcohol consumption, physical activity, income level, BMI, hypertension, and total cholesterol level. In addition, the risk of AD and VD in participants in the 4th quartile of the TyG index increased 12% and 18%, respectively, compared to that in participants in the 1st quartile of TyG index which increased across increasing TyG index quartiles (all *p* for trend < 0.001, Table 2) irrespective of several potential confounders that included age, sex, smoking status, alcohol consumption, physical activity, low income, BMI, hypertension, and total cholesterol level. In addition, the present results were consistent across sensitivity and subgroup analyses. This nationwide longitudinal observational study with 5,586,048 participants is one of the largest studies in which the association between TyG index and dementia was shown and one of the largest studies in which the association between insulin resistance and dementia was investigated.

Over the last decades, the effects of insulin resistance in patients with dementia have been investigated in several studies; however, insulin resistance was mainly measured using the homeostasis model assessment of insulin resistance (HOMA-IR) index. In the Rotterdam study with 3139 participants, a higher risk of AD was associated with insulin levels and insulin resistance based on the HOMA-IR index, although only within 3 years from baseline and not after 3 years [15]. In the PERF Study (*n* = 1759), an observational, prospective cohort study of Danish postmenopausal women, the risk of cognitive dysfunction, which was assessed using two short cognitive screening tools, increased between 8 and 10% for every unit increase in the HOMA-IR index scale [16]. In addition, in the recent CAIDE study of 269 dementia-free individuals 65–79 years of age, an increase in HOMA-IR values was associated with worse cognitive performance 7 years later in elderly individuals without dementia [17]. Conversely, the association between dementia and insulin resistance was not demonstrated in several studies. In the Uppsala Longitudinal Study of Adult Men (*n* = 1125), the risk of AD and VD showed no association with insulin sensitivity based on the euglycemic insulin clamp method, the gold standard for assessing insulin resistance, after a median follow-up of 12 years [18]. The inconsistencies across studies may be explained by heterogeneities in study designs, populations, and variability in cognitive assessment methods and insulin resistance. The present study is the first in which a positive relationship was observed between TyG index quartile, another surrogate marker of insulin resistance, and the risk of dementia in a large population without using the HOMA-IR. This study’s results support the previous positive results based on the HOMA-IR index with respect to other aspects with the TyG index in a large population. In addition, even in various subgroup analyses, the relationship between the TyG index and dementia was consistent. Subgroup analysis showed a stronger association between TyG index and all-cause dementia, AD, and VD in younger participants (< 65 years old, Fig. 2). And the association between the TyG index and all-cause dementia, AD, and VD was weaker in participants with an insulin resistance phenotype that included hypertension, obesity, no regular exercise, and abdominal obesity (Fig. 2). However, this finding was not observed in subgroup by diabetes and dyslipidemia, as these might be caused by selection bias. We excluded the participants who took anti-diabetic medications or lipid-lowering medications from this study, so further studies are needed. When comparing the association between AD and the TyG index, the association between VD and the TyG index was stronger in current smokers (*p* for interaction = 0.036), which was not related to insulin resistance but is a well-established risk factor for cardiovascular disease. Further, the association between VD and the TyG index was weaker in obese participants (BMI ≥ 25 kg/m^2^) which was also associated with insulin resistance and well-established risk factors for cardiovascular disease, when comparing the association between AD and the TyG index.

A number of various complexity measures have emerged for insulin resistance. The hyperinsulinemic euglycemic clamp is considered the reference method for the measurement of insulin resistance and the gold standard. However, the hyperinsulinemic euglycemic clamp is a costly, time-consuming, and invasive method that requires a skilled operator; therefore, surrogate measures, especially for large-scale epidemiological studies, are needed. Some simple indirect methods, such as HOMA-IR [19] and the quantitative insulin sensitivity check index (QUICKI) [20], have been proposed. These two methods use fasting insulin and glucose concentrations to measure insulin resistance, and the results correlate well with the values reported in clamp studies. In addition, an increase in HOMA-IR and QUICKI values has been shown to be associated with an increased incidence and risk of diabetes and future cardiovascular events [21–23]. However, these models have higher coefficients of variation due to the pulsatile pattern of insulin secretion and cannot detect early stage insulin resistance [24, 25]. A recent study was conducted to find biomarkers that identify early stage insulin resistance. Based on the rationale that effects of insulin on lipolysis occur at lower levels than for glucose metabolism, the TyG index, the product derived from fasting blood glucose and triglyceride levels, has been suggested as a simple alternative surrogate marker of insulin resistance [11]. Guerrero-Romero et al. reported the TyG index, regardless of varying glucose tolerance levels and body weights, inversely correlates with total glucose metabolism rate, which is an insulin sensitivity marker determined using the euglycemic-hyperinsulinemic clamp test as the gold standard method [12]. In another study with 82 Brazilian participants, the TyG index showed better performance for predicting insulin resistance using the hyperglycemic clamp test compared with the HOMA2-IR index [26]. Reportedly, individuals in the 4th quartile of the baseline TyG index had a 4-fold higher risk of developing diabetes compared with the 1st quartile, and the TyG index was superior to the HOMA-IR in predicting the risk of incident diabetes [27]. Sánchez-Íñigo et al. reported that patients in the 4th quartile of the TyG index had 2.32 times (95% CI 1.65–3.26) higher risk of developing cardiovascular disease compared with patients in the 1st quartile after adjusting for confounding factors [28]. Furthermore, unlike the HOMA-IR and QUICKI, insulin is not included in the TyG index [29] and this simplicity has practical consequences such as better accessibility and lower cost that may be important in a large-scale population study.

There are several possible mechanisms to explain the role of insulin resistance in the development of dementia. First, cerebrovascular disease, a consequence of insulin resistance, can induce the development and progression of VD and AD by causing multifocal ischemic lesions, and has been shown to predict the development or progression of cognitive decline in several clinical studies [30]. Second, the alteration of brain insulin signaling may be due to insulin resistance associated with induction of cognitive impairments and neurodegeneration [31]. In a previous study, intranasal insulin therapy improved cognitive function and slowed cognitive decline [32] and the insulin sensitizer, pioglitazone, showed time- and dose-dependent effects for preventing the development of dementia in patients with diabetes [33]. Third, insulin resistance-related syndromes, such as diabetes and dyslipidemia, are well-known risk factors for dementia. In a recent meta-analysis that included 19 community-based studies, type 2 diabetes patients had dementia 1.6 times more often than patients without type 2 diabetes [34]. In another meta-analysis of 17 studies, high plasma cholesterol in mid-life was associated with a 2.14-fold increased risk of AD dementia but not in late life [35]. In the present study, increasing TyG index was associated with increasing risk of dementia in the population with diabetes or dyslipidemia, indicating insulin resistance may cause dementia independently of diabetes and dyslipidemia. In addition, the increasing TyG index was associated with increasing risk of dementia in the population with other cerebrovascular risk factors that included age, smoking status, low physical activity, obesity, diabetes, and dyslipidemia. The results of the present study provide evidence that insulin resistance is associated with the development of dementia separate from other insulin resistance-related diseases. In addition, we also assessed the association between each individual triglyceride and glucose, and dementia. High triglycerides and glucose were also associated with higher risk of dementia. The analysis by quartiles of triglyceride showed higher quartiles were associated with a higher risk of all-cause dementia (2nd, 3rd, and 4th quartiles), AD (3rd and 4th quartiles), and VD (3rd and 4th quartiles) compared to the 1st quartile (Supplementary Table 1). Only the 4th glucose quartile was associated with a higher risk of all-cause dementia and AD, but not VD compared to the 1st quartile (Supplementary Table 1). However, HR comparison between glucose, triglyceride, and TyG index was inadequate, as each participant in the 1st glucose and triglyceride quartiles or TyG index was different. Therefore, we conducted another subgroup analysis to assess the association between TyG index and dementia by triglyceride and glucose quartiles (Supplementary Fig. 1). Increasing TyG index quartile was significantly associated an increasing risk for all-cause dementia, AD, and VD regardless of triglyceride and glucose quartile except for the 1st triglyceride quartile (this may be due to an exceedingly low incidence of dementia, Supplementary Fig. 1). Calculating the TyG index is inconvenient in clinical practice, and the effect size for the TyG index in our study is small; therefore, the direct usefulness of the TyG index in clinical practice is doubtable. However, our study results also suggest that patients with high glucose and/or triglyceride levels may be considered at high risk for dementia and should be considered for treatment to reduce insulin resistance in order to reduce the risk of dementia. Further, in real clinical practice, the TyG index could be a considerable component to evaluate dementia risk.

### Limitations

The strength of the present study is the use of a large-scale nationwide database representing the entire Korean population. However, the present study had several limitations. First, the retrospective observational study design had inherent limitations. Although the analyses were adjusted for most demographic and clinical variables available, some unidentified parameters could have affected the results. Second, dementia was defined based on the claim data; therefore, differences with the real diagnoses may exist. Furthermore, in clinical practice, the dementia diagnosis is occasionally difficult, which can cause misclassification or information bias. To overcome these problems, the outcomes were defined by combining the diagnosis and the prescription records. In addition, the proportion of AD and VD dementia cases (74.3% and 12.5%, respectively) in the present study were similar to previous reports [36, 37]. Third, data on the severity of dementia were not available; thus, the association between insulin resistance and severity and progression of dementia could not be evaluated. Fourth, insulin resistance was not directly measured. Although the association between the TyG index and insulin resistance was shown in a previous study, discrepancies may exist. Thus, the insulin resistance of 5,586,048 participants could not be realistically measured. Lastly, although the association between the TyG index and dementia was statistically significant, the effect size of the TyG index on dementia was relatively small. This indicates that the TyG index might only be a minor predictor of dementia, but there was also the possibility of underestimating the association between the TyG index and dementia, because age and insulin resistance showed collinearity which could be a cause of the statistical underestimation for risk seen in the multivariate-adjusted model. However, the risk of dementia was robustly increased across TyG index quartiles even after adjusting for age, sex, and other covariates, and in subgroup analysis, younger participants (below 65 years old) showed a stronger association compared to older participants (65 and over years old, *p* for interaction < 0.001).

## Conclusions

During a median follow-up of 7.21 years with 5,586,048 participants, higher quartiles of the TyG index were associated with a higher risk of dementia, indicating the TyG index, as a surrogate marker of insulin resistance, may be an independent but minor predictor of dementia development, due to its small effect size. Although a causal interpretation of our findings cannot be made due to the observational study design with data obtained from the information recorded in a claims database, targeting and treating insulin resistance prior to the development of dementia may lead to reductions in dementia-related public health burdens.

## Supplementary Information

**Additional file 1: Supplementary Table 1**. Risk of dementia, AD, and VD based on the quartiles of triglyceride, glucose, and TyG index. **Supplementary Fig. 1**. Risk of dementia in AD and VD subgroups based on increasing TyG index quartiles with the quartiles for glucose and triglyceride adjusted for age, sex, smoking status, alcohol consumption, physical activity, low income, body mass index, hypertension, and total cholesterol level. HR, hazard ratio; CI, confidence interval.

## Data Availability

The datasets generated during and/or analyzed during the current study are available in the National Health Insurance Sharing Service (https://nhiss.nhis.or.kr/).

## Abbreviations

AD: Alzheimer’s disease
BP: Blood pressure
BMI: Body mass index
CKD: Chronic kidney disease
CI: Confidence interval
HR: Hazard ratio
HDL: High-density lipoprotein
HOMA-IR: Homeostasis model assessment of insulin resistance
LDL: Low-density lipoprotein
NHID: National Health Information Database
QUICKI: Quantitative insulin sensitivity check index
TyG: Triglyceride glucose
VD: Vascular dementia
