# Supplementary material for: The insulin resistance by triglyceride glucose index and risk for dementia: population-based study
Source: Alzheimers Res Ther. 2021 Jan 5;13:9. doi: 10.1186/s13195-020-00758-4 (PMC7786939; doi:10.1186/s13195-020-00758-4)

Supplementary Table 1. Risk of dementia, AD, and VD based on the quartiles of triglyceride, glucose, and TyG index.

|  | Events (n) | Duration  (person-years) | Incidence rate  (per 1,000 person-years) | Unadjusted | MODEL 1 | MODEL 2 |
| --- | --- | --- | --- | --- | --- | --- |
| All-cause dementia | | |  |  |  |  |
| Triglyceride | |  |  |  |  |  |
| Q1 | 27810 | 10125259.16 | 2.75 | 1(ref.) | 1(ref.) | 1(ref.) |
| Q2 | 33361 | 10078936.74 | 3.31 | 1.21(1.19,1.22) | 1.02(1.00,1.03) | 1.02(1.01,1.04) |
| Q3 | 38819 | 10067491.58 | 3.86 | 1.40(1.38,1.42) | 1.05(1.03,1.06) | 1.06(1.04,1.08) |
| Q4 | 42724 | 10000071.13 | 4.27 | 1.55(1.53,1.58) | 1.08(1.07,1.10) | 1.10(1.09,1.12) |
| Glucose |  |  |  |  |  |  |
| Q1 | 32015 | 10360754.21 | 3.09 | 1(ref.) | 1(ref.) | 1(ref.) |
| Q2 | 32663 | 10231622.68 | 3.19 | 1.03(1.02,1.05) | 0.98(0.97,1.00) | 0.99(0.98,1.01) |
| Q3 | 33739 | 9690659.47 | 3.48 | 1.13(1.11,1.15) | 0.99(0.98,1.01) | 1.01(0.99,1.03) |
| Q4 | 44297 | 9988722.25 | 4.43 | 1.44(1.42,1.46) | 1.06(1.04,1.07) | 1.07(1.06,1.09) |
| TyG index | |  |  |  |  |  |
| Q1 | 26613 | 10089076.34 | 2.64 | 1(ref.) | 1(ref.) | 1(ref.) |
| Q2 | 32819 | 10074648.59 | 3.26 | 1.23(1.22,1.26) | 1.03(1.01,1.04) | 1.04(1.02,1.05) |
| Q3 | 38606 | 10066646.27 | 3.84 | 1.45(1.43,1.48) | 1.05(1.04,1.07) | 1.07(1.05,1.09) |
| Q4 | 44676 | 10041387.42 | 4.45 | 1.69(1.66,1.71) | 1.11(1.09,1.13) | 1.14(1.12,1.16) |
| AD |  |  |  |  |  |  |
| Triglyceride | |  |  |  |  |  |
| Q1 | 20544 | 10125259.16 | 2.03 | 1(ref.) | 1(ref.) | 1(ref.) |
| Q2 | 24741 | 10078936.74 | 2.45 | 1.21(1.19,1.23) | 1.01(0.99,1.03) | 1.02(1.00,1.04) |
| Q3 | 28875 | 10067491.58 | 2.87 | 1.41(1.39,1.44) | 1.03(1.01,1.04) | 1.05(1.03,1.07) |
| Q4 | 31858 | 10000071.13 | 3.19 | 1.57(1.54,1.60) | 1.06(1.04,1.07) | 1.09(1.07,1.11) |
| Glucose |  |  |  |  |  |  |
| Q1 | 23751 | 10360754.21 | 2.29 | 1(ref.) | 1(ref.) | 1(ref.) |
| Q2 | 24220 | 10231622.68 | 2.37 | 1.03(1.02,1.05) | 0.98(0.97,1.00) | 0.99(0.98,1.01) |
| Q3 | 25159 | 9690659.47 | 2.60 | 1.14(1.12,1.16) | 1.00(0.98,1.02) | 1.02(1.00,1.03) |
| Q4 | 32888 | 9988722.25 | 3.29 | 1.44(1.42,1.47) | 1.05(1.03,1.07) | 1.07(1.05,1.09) |
| TyG index | |  |  |  |  |  |
| Q1 | 19705 | 10089076.34 | 1.95 | 1(ref.) | 1(ref.) | 1(ref.) |
| Q2 | 24354 | 10074648.59 | 2.42 | 1.24(1.21,1.26) | 1.02(1.00,1.03) | 1.03(1.01,1.05) |
| Q3 | 28661 | 10066646.27 | 2.85 | 1.46(1.43,1.48) | 1.03(1.01,1.05) | 1.05(1.03,1.07) |
| Q4 | 33298 | 10041387.42 | 3.32 | 1.70(1.67,1.73) | 1.07(1.06,1.09) | 1.12(1.09,1.14) |
| VD |  |  |  |  |  |  |
| Triglyceride | |  |  |  |  |  |
| Q1 | 3513 | 10125259.16 | 0.35 | 1(ref.) | 1(ref.) | 1(ref.) |
| Q2 | 4167 | 10078936.74 | 0.41 | 1.19(1.14,1.25) | 1.06(1.01,1.11) | 1.03(0.98,1.08) |
| Q3 | 4803 | 10067491.58 | 0.48 | 1.37(1.32,1.44) | 1.12(1.07,1.17) | 1.08(1.03,1.13) |
| Q4 | 5317 | 10000071.13 | 0.53 | 1.53(1.47,1.60) | 1.21(1.16,1.26) | 1.13(1.08,1.19) |
| Glucose |  |  |  |  |  |  |
| Q1 | 4101 | 10360754.21 | 0.40 | 1(ref.) | 1(ref.) | 1(ref.) |
| Q2 | 4119 | 10231622.68 | 0.40 | 1.02(0.98,1.06) | 0.97(0.93,1.01) | 0.97(0.93,1.01) |
| Q3 | 4091 | 9690659.47 | 0.42 | 1.07(1.02,1.12) | 0.95(0.91,0.99) | 0.95(0.91,0.99) |
| Q4 | 5489 | 9988722.25 | 0.55 | 1.39(1.34,1.45) | 1.07(1.03,1.11) | 1.03(0.99,1.08) |
| TyG index | |  |  |  |  |  |
| Q1 | 3341 | 10089076.34 | 0.33 | 1(ref.) | 1(ref.) | 1(ref.) |
| Q2 | 4110 | 10074648.59 | 0.41 | 1.23(1.18,1.29) | 1.08(1.03,1.13) | 1.05(1.01,1.10) |
| Q3 | 4779 | 10066646.27 | 0.47 | 1.43(1.37,1.50) | 1.14(1.09,1.19) | 1.09(1.05,1.15) |
| Q4 | 5570 | 10041387.42 | 0.55 | 1.68(1.61,1.75) | 1.25(1.20,1.31) | 1.18(1.12,1.23) |

AD, Alzheimer’s disease; VD, vascular dementia; TyG, triglyceride glucose index; HR, hazard ratio; CI, confidence interval

MODEL 1: Age, sex

MODEL 2: Age, sex, smoking status, alcohol consumption, physical activity, low income, body mass index, hypertension, total cholesterol level

Supplementary Figure 1. Risk of dementia in AD and VD subgroups based on increasing TyG index quartiles with the quartiles for glucose and triglyceride adjusted for age, sex, smoking status, alcohol consumption, physical activity, low income, body mass index, hypertension, and total cholesterol level. HR, hazard ratio; CI, confidence interval


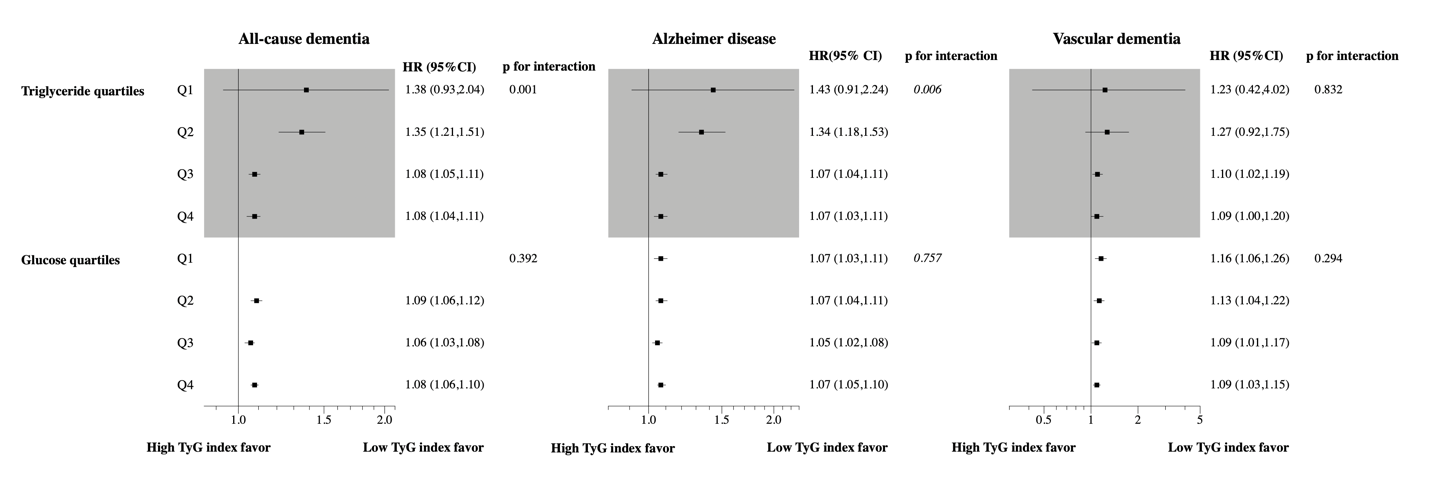

Supplement: Supplementary file 1 — Additional file 1: Supplementary Table 1. Risk of dementia, AD, and VD based on the quartiles of triglyceride, glucose, and TyG index. Supplementary Fig. 1. Risk of dementia in AD and VD subgroups based on increasing TyG index quartiles with the quartiles for glucose and triglyceride adjusted for age, sex, smoking status, alcohol consumption, physical activity, low income, body mass index, hypertension, and total cholesterol level. HR, hazard ratio; CI, confidence interval. [file 13195_2020_758_MOESM1_ESM.docx]
